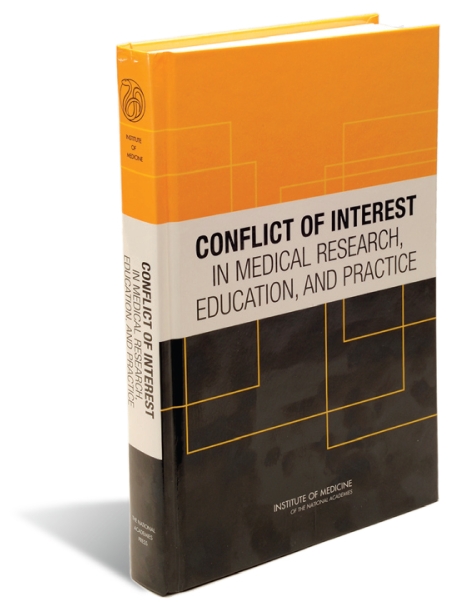# Conflict of Interest in Medical Research, Education, and Practice

**Published:** 2010-02

**Authors:** David B. Resnik

**Affiliations:** David B. Resnik is a bioethicist and chair of the Institutional Review Board at the National Institute of Environmental Health Sciences, National Institutes of Health. He has published 7 books and 150 articles on ethical, philosophical, and legal issues in science, medicine, and technology.

Concerns about the potentially corrupting influence of private industry on medical research, education, and practice have been growing since the 1970s, when the U.S. Congress passed the first anti-kickback law applying to medicine. Since then, Congress has banned self-referrals in medicine, enacted laws that regulate the commercialization of publicly funded biomedical research, and held hearings on conflicts of interest in biomedicine. Government agencies have issued policies concerning disclosure of financial interests in research, professional associations have published guidelines pertaining to financial relationships with industry, and scientific journals have developed conflict of interest policies.

The Institute of Medicine (IOM)’s *Conflict of Interest in Medical Research, Education, and Practice* makes an important contribution to the continuing debate about financial conflicts of interest in medicine. This report reviews empirical studies, analytic essays, regulations, and guidance documents concerning conflicts of interest in medical research and makes recommendations for researchers, educators, physicians, institutions, corporations, and government agencies. The report consists of nine chapters as well as appendices that examine particular topics in greater depth, such as the relevance of psychological research on subconscious biases to conflict of interest policies, and a comparative analysis of conflicts of interest in nonmedical professions.

To draft this report, the IOM assembled experts from various fields including medicine, public health, law, health policy, ethics, philosophy, and economics. The authors acknowledge that collaboration with industry is a double-edged sword: Collaboration can produce important benefits, but it can compromise the integrity of professional judgment and erode the public’s trust in medicine. In crafting their recommendations, the authors strike a balance between protecting medical research, education, and practice from the harmful effects of industry collaboration and avoiding unnecessary administrative burdens that could undermine the benefits of collaboration. They make judicious use of empirical evidence to support their conclusions and recommendations. When evidence was lacking, the authors developed arguments based on their own experience and judgment as well as different approaches found in the literature.

The IOM report makes 16 recommendations for reforming medicine, some of which would require radical changes. For example, the report recommends that all academic medical centers, teaching hospitals, and training sites prohibit faculty, students, residents, and fellows from accepting gifts from pharmaceutical, biotechnology, or medical device companies, except in some circumstances, and not allow drug and medical sales representatives to have access to faculty, except by invitation in specified situations. The IOM report also recommends that academic medical centers, hospitals, and training sites develop a new system of funding continuing medical education that is free from industry influence; that research institutions prohibit investigators from conducting research with human subjects if they have a significant financial interest related to a product that would be affected by the research, except when the investigator’s participation is essential for conducting the research; and that physicians in private practice refrain from accepting items of value from pharmaceutical, biotechnology, or medical device companies.

The report also contains a conceptual framework that provides a foundation for policy recommendations. This includes a definition of conflict of interest (“a set of circumstances that creates a risk that professional judgment or actions regarding a primary interest will be unduly influenced by a secondary interest”) and criteria for assessing conflict of interest policies, such as proportionality (does the policy focus on the most important conflicts and is the policy practical?), transparency (is the policy understandable to those to whom it applies?), accountability (does the policy have enforcement procedures?), and fairness (does the policy apply equally to all relevant people in similar situations?). The authors also discuss problems with interpreting and applying the notion of an apparent conflict of interest.

This comprehensive and insightful report will undoubtedly have considerable influence over research, scholarship, and policy development for years to come. It is highly recommended reading for physicians, medical researchers, and medical educators.

## Figures and Tables

**Figure:**